# Prevalence of Chronic Diseases in Residents of the Qassim Region, Saudi Arabia: A Community-Based Cross-Sectional Study

**DOI:** 10.7759/cureus.81735

**Published:** 2025-04-04

**Authors:** Sundus H Aldakhil, Yara Alromaih, Norah Aljuaylan, Ghadi A Alkhalaf, Amani Alharbi, Jolan S Alsaud

**Affiliations:** 1 Research and Studies Unit, The Friends of Patients Association in Unayzah Governorate, Unayzah, SAU; 2 Family Medicine, Qassim Health Cluster, Qassim, SAU

**Keywords:** cardiovascular disease, diabetes mellitus, epidemiology, hypertension, obesity, prevalence, risk factors

## Abstract

Background

Chronic diseases (CDs) pose a significant burden on healthcare systems worldwide. Despite extensive global research, specific data on the prevalent CDs in the Qassim Region of Saudi Arabia remain limited. Therefore, we analyzed the prevalent CDs among residents of the Qassim region based on age- and sex-related differences, to provide useful data for formulating public health policies and health promotion initiatives.

Methods

A cross-sectional study was conducted between August and September 2024, using a structured questionnaire administered to a convenience sample of 387 adults (≥18 years) from multiple public locations in Qassim. Descriptive statistics, Chi-square tests, and multivariate logistic regression analyses were performed using SPSS v.25.

Results

Among the 387 participants, 154 (39.7%) had at least one CD. The most prevalent conditions were diabetes mellitus (DM; n=70, 27.67%) and hypertension (n=60, 23.72%), with significantly higher rates among females (n=46, 26.29%). Obesity was more prevalent in males (p = 0.05). The prevalence of multimorbidity increased significantly with age (p < 0.001).

Conclusion

Our findings provide valuable insights into the prevalence of CDs in the Qassim region, Saudi Arabia, and combined with existing literature, emphasize the necessity of continued efforts to prevent and manage these chronic conditions to promote the health and well-being of the community in the Qassim region.

## Introduction

Chronic diseases (CDs) are persistent conditions that often require continuous medical treatment and can significantly limit daily activities. CDs, including cardiovascular diseases, diabetes mellitus (DM), respiratory diseases, and cancer, are prevalent worldwide [1]. In both the United States and globally, CDs are the leading causes of morbidity and disability, impacting individuals across all age groups [1,2]. The WHO refers to CDs as the 'silent pandemic,' given that they account for approximately 38 million deaths annually, representing 63% of global mortality rates [3,4]. Among individuals aged over 70 years old, the leading causes of death worldwide in 2019 included DM, ischemic heart disease, stroke, chronic obstructive pulmonary disease, Alzheimer’s disease, lower respiratory tract infections, and chronic kidney disease [5].

The global cost of CDs is projected to reach $47 trillion by 2030. In Saudi Arabia (SA), the annual cost associated with CDs is expected to be $24.4 billion [2,6]. The economic burden of CDs includes medical expenses and loss of productivity due to illness, which creates substantial challenges for affected individuals and their families [7].

Among the Middle Eastern countries, SA reports one of the highest incidences of CDs, with a prevalence rate of 32.15% [8], posing significant health challenges. DM, hypertension, and obesity are the leading causes of morbidity [9,10]. The rise in the prevalence of CDs adversely impacts the quality of life of patients and increases overall healthcare costs [5]. Healthcare services focus on enhancing patient care and minimizing morbidity and mortality, as well as strive to elevate the Health-related Quality of Life (HRQoL) for individuals with chronic conditions [11,12]. Alsaud JS et al. conducted a study on patients with thyroid cancer in SA and reported the significant impact of the disease on social functioning, thereby highlighting the importance of addressing quality of life alongside medical care [13]. Other countries in the region, such as Qatar and Kuwait, also face rising rates of conditions like diabetes and hypertension, particularly among older populations [14,15].

While advances in healthcare and healthy lifestyle initiatives are vital in improving health outcomes, CDs remain a critical challenge [16]. Reducing risk factors associated with CDs is essential to prevent associated morbidity and premature death [17]. In SA, risk factors including high body mass index and elevated blood sugar levels are common; these are compounded by demographic shifts that strain the healthcare system [17,18]. Understanding these patterns and factors is crucial for developing effective health policies as countries transition from infectious to chronic illnesses [6,17].

Despite previous studies on CDs worldwide and regionally, rapid growth of the population and the ongoing change in demographics necessitate a reevaluation of these findings. Analyzing CDs rates in Qassim Region provides insights into regional health and can guide focused public health initiatives and healthcare strategies tailored to the specific health needs of the community [18]. Accordingly, this study was conducted to assess the prevalence of chronic diseases in adults) in the Qassim region, SA.

Secondary objectives were also defined: to analyze the association between age and the prevalence of CDs, to compare CD prevalence between males and females, and to evaluate trends of common CDs such as diabetes, hypertension, and obesity.

## Materials and methods

Study design and sampling

​A cross-sectional study was conducted from August 2024 to September 2024 in multiple public locations across Qassim. This survey was carried out by the Patients Friends Association in Unayzah Governorate, a charitable association that assists patients according to their needs and engages in health promotion activities in the Qassim region. Participants were selected using a non-probability convenience sampling technique. While this approach was necessary due to logistical constraints, efforts were made to enhance representativeness by selecting individuals from diverse demographic backgrounds, ensuring adequate distribution across different age groups and sexes.

Study location and data collection

The study was conducted among adults in various cities and public locations within the Qassim region of SA, including health centers, malls, public parks, and awareness exhibitions. Participants were recruited based on their presence in these locations, with efforts to enhance representativeness by selecting diverse public venues across different cities in the Qassim region. The study questionnaire was distributed by trained healthcare providers who approached the participants, explained the objectives of the questionnaire, and obtained written informed consent. Participation was voluntary.

Study tool

A structured, self-administered questionnaire was developed based on an extensive literature review and expert consultations. It consisted of two sections: demographic data including age, sex, and place of residence; and health information regarding the presence of CDs and the type of disease. To ensure the content validity, comprehensibility, and relevance of the questionnaire, we invited two experts in public health outside the team to review it. Additionally, a pilot test was conducted among 30 participants to assess clarity and reliability, yielding a Cronbach’s alpha (α) of 0.82, indicating good internal consistency. The questionnaire was developed and distributed in Arabic, and the responses were translated into English by a language expert.

Inclusion and exclusion criteria

We included adults (≥18 years) residing in Qassim and individuals capable of completing the questionnaire independently or with assistance.

We excluded participants who did not fully complete the questionnaire and non-residents of Qassim.

Sample size

The sample size was calculated using Cochran’s formula:

N = \frac{{Z^2 \times P (1-P)}}{{d^2}} 

where: Z = 1.96 (for a 95% confidence level), P = 50% (since no prior prevalence estimate for CDs in Qassim was available), and d = 5% (margin of error).

Based on these calculations, the minimum required sample size was 384 participants. However, to improve statistical power and ensure broad representation, a total of 600 participants were included in the study, of whom 384 completed the questionnaire, yielding a response rate of approximately 64%. This rate is comparable to similar community-based studies, ensuring adequate representation of the target population.

Data analysis

Statistical analysis was conducted using SPSS v.25 (IBM Corp., Armonk, NY, US). Excel was used to extract and summarize questionnaire data. Data were then encoded and exported from the Excel database. Descriptive statistics are presented as means and percentages. The chi-square test was applied to examine associations between categorical variables, while logistic regression analysis was used to control for potential confounders. A p-value < 0.05 was considered statistically significant. All CIs reported in this study were derived from the original dataset using SPSS v.25. The calculations were performed based on logistic regression and Chi-square tests to ensure statistical accuracy.

Bias reduction measures**

To minimize potential biases, diverse data collection locations were set up across multiple cities in Qassim to improve representativeness. Participants were recruited from different age groups and both sexes to enhance sample diversity and ensure a more representative distribution. Self-reported data were validated by instructing participants on the importance of accurate reporting.

Ethical considerations

Ethical approval was obtained from the Regional Research Ethics Committee of Qassim Province, reference number (607-46-1075). The confidentiality of participant responses and their right to withdraw from the study were assured.

## Results

Demographic characteristics of participants

Our findings revealed a diverse age and sex distribution among the 387 participants. Overall, n=116, 30.0% of individuals were over 50 years old, and a combined n=185, 47.9% in the 41-50 and >50 age ranges. In terms of sex distribution, women constituted a substantial majority at n=283, 73.1%, versus n=104, 26.9% for men, indicating a potential sex-specific disparity in disease patterns (Table 1).

**Table 1:** Demographic characteristics of the study population (N=387).

Variable	N (%)
Age groups	
18-20 years	17 (4.39)
20-30 years	101 (26.1)
31-40 years	84 (21.7)
41-50 years	69 (17.9)
>50 years	116 (30)
Sex	
Male	104 (26.9)
Female	283 (73.1)

**Figure 1:**
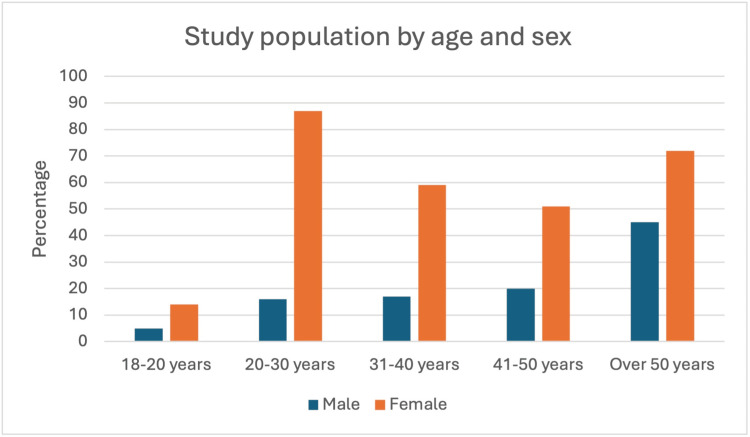
Age- and sex-specific distribution among study participants in the Qassim Region, Saudi Arabia.

The data from Figure 1 depicting the distribution of men and women across various age groups illustrate a consistent sex disparity throughout the lifespan. Women are more prevalent than men in all age brackets, with the largest difference observed in the 20-30 years age group, where data from 86 women are compared with that of only 15 men. This pattern persists across the remaining age groups, with women consistently outnumbering men by a substantial margin. For example, in the over 50 years category, data from 71 women were compared with that of 45 men.

Prevalence of CDs by condition

Among 387 respondents from the Qassim Region, the prevalence of various conditions among the 154 individuals with CDs revealed that diabetes was the most prevalent condition, affecting n=70, 27.67% of this group, followed by hypertension at n=60, 23.72% and hyperlipidemia at n=34, 13.44%. The prevalence of obesity and cardiovascular disease were also high, impacting approximately n=24, 9.49% and n=22, 8.70% of respondents, respectively. Although not prevalent, conditions such as breast cancer (n=4, 1.58%), ovarian cancer (n=2, 0.79%), multiple sclerosis (n=2, 0.79%), and hypothyroidism (n=12, 4.74%), were observed, each affecting a relatively small percentage of individuals within the study population. These findings highlight the most pressing chronic health issues in this region (Table 2).

**Table 2:** Prevalence of chronic diseases among the study population. # Multiple response. CDs: Chronic diseases.

Chronic Disease #	N (%)
Participants with CDs	154 (39.7%)
Diabetes Mellitus	70 (27.67%)
Obesity	24 (9.49%)
Hypertension	60 (23.72%)
Chronic kidney disease	7 (2.77%)
Cardiovascular disease	22 (8.70%)
Hyperlipidemia	34 (13.44%)
Asthma	10 (3.95%)
Breast cancer	4 (1.58%)
Thyroid cancer	1 (0.40%)
Cervical cancer	1 (0.40%)
Prostate enlargement	4 (1.58%)
Ovarian cancer	2 (0.79%)
Multiple sclerosis	2 (0.79%)
Hypothyroidism	12 (4.74%)

Analysis of CD prevalence by age

Table 3 illustrates the prevalence of various CDs across different age groups. We observed that the prevalence of diabetes, obesity, hypertension, and hyperlipidemia significantly increased with age, particularly after 50 years, with diabetes reaching n=45, 17.79% in those over 50 years of age (all P <0.05). Conversely, the prevalence of chronic kidney disease and cardiovascular disease was relatively low across age groups, with chronic kidney disease having a prevalence of n=1, 0.40% in those over 50 years of age, and cardiovascular disease being more prevalent but still relatively low in all age groups. The prevalence of hyperlipidemia rises significantly with age, from n=0, 0% in those under 20 years of age to n=25, 9.88% in those over 50 years of age (P = 0.002; 95% CI = 2.44-5.85). These findings suggest that older individuals are 2.44-5.85 times more likely to have the condition. We also observed a trend in the prevalence of various cancer types. For instance, thyroid cancer was observed in the 20-30 years age group but with a p-value of 0.05, suggesting a borderline statistical significance. Ovarian cancer, observed only in those over 50 years, has a p-value of 0.04, indicating a statistically significant age-related increase. Additionally, conditions such as hypothyroidism are also age-related, with a prevalence of n=3, 1.19% in the 31-40 years group and n=5, 1.98% in those over 50 (p = 0.004), which highlights a significant age-dependent variation (Table 3).

**Table 3:** Prevalence of chronic diseases according to age. * Significant at p < 0.05 level.

	Age groups					P-value	95% CI
Disease	18-20 years	20-30 years	31-40 years	41-50 years	Over 50 years		
	N (%)	N (%)	N (%)	N (%)	N (%)		
Diabetes	3 (1.19)	5 (1.98)	5 (1.98)	12 (4.74)	45 (17.79)	0.002*	3.55-5.24
Obesity	0 (0.00)	2 (0.79)	5 (1.98)	3 (1.19)	14 (5.53)	0.003*	2.49-4.77
Hypertension	0 (0.0)	2 (0.79)	5 (1.98)	15 (5.93)	38 (15.02)	0.001*	3.88-4.56
Chronic kidney disease	0 (0.0)	2 (0.79)	2 (0.79)	2 (0.79)	1 (0.40)	0.04*	1.44-2.88
Cardiovascular disease	0 (0.00)	4 (1.58)	5 (1.98)	5 (1.98)	8 (3.16)	0.04*	3.59-6.41
Hyperlipidemia	0 (0.0)	2 (0.79)	2 (0.79)	5 (1.98)	25 (9.88)	0.002*	2.44-5.85
Asthma	1 (0.40)	2 (0.79)	2 (0.79)	1 (0.40)	4 (1.58)	0.03*	3.68-5.56
Breast cancer	0 (0.0)	0 (0.0)	1 (0.40)	3 (1.19)	0 (0.0)	0.05	1.88-3.88
Thyroid cancer	0 (0.0)	1 (0.40)	0 (0.0)	0 (0.0)	0 (0.0)	0.05	1.53-3.87
Cervical cancer	0 (0.0)	0 (0.0)	0 (0.0)	1 (0.40)	0 (0.0)	0.05	1.23-2.55
Prostate enlargement	0 (0.0)	0 (0.0)	0 (0.0)	0 (0.0)	4 (1.58)	0.023*	1.98-2.54
Ovarian cancer	0 (0.0)	0 (0.0)	0 (0.0)	0 (0.0)	2 (0.79)	0.04*	1.69-2.71
Multiple sclerosis	0 (0.0)	0 (0.0)	1(0.40)	1 (0.40)	0 (0.0)	0.004*	2.66-5.36
Hypothyroidism	0 (0.00)	2 (0.79)	3(1.19)	2 (0.79)	5 (1.98)	0.003*	2.87-4.89
People with multimorbidity							
No chronic disease	13 (3.36)	85 (21.96)	64 (16.54)	38 (9.82)	33 (8.53)	0.002*	1.06-3.86
One chronic disease	4 (1.03)	14 (3.62)	15 (3.88)	17 (4.39)	33 (8.53)	0.03*	2.86-5.22
Two chronic diseases	00 (00)	2 (0.52)	4 (1.03)	7 (1.81)	22 (5.68)	0.001*	1.98-4.98
≥Three chronic diseases	00 (00)	00 (00)	1 (0.26)	7 (1.81)	28 (7.24)	0.04*	3.88-6.84

Analysis of CD prevalence by sex

Table 4 illustrates significant sex-related differences in the prevalence of various CDs. We observed that diabetes and hypertension were more prevalent in women compared with men (n=56, 32% and n=46, 26.29%, respectively, vs. n=14, 17.95% for both conditions; p = 0.002 and 0.003, respectively). Conversely, we observed a significantly disproportionate prevalence of obesity among men compared with that in women (n=16, 20.51% vs. n=8, 4.57%; p = 0.05). The prevalence of cardiovascular disease was slightly but not significantly different between women and men (n=13, 7.43% vs. n=9, 10.39%; p = 0.02).The sex-specific prevalence of conditions, such as chronic kidney disease, asthma, and hypothyroidism, was less pronounced but statistically significant. Specific cancers such as breast, ovarian, and thyroid cancer affected n = 4, 2.29%, n=2, 1.14% and n=1, 0.57% of women, respectively. Prostate-related conditions such as prostate enlargement observed in n=4, 3.27% of men (p = 0.004). The proportion of individuals without CDs was significantly higher among women than among men (n=191, 67.49% vs. n=42, 40.38%; p < 0.001), thereby highlighting the need for sex-specific approaches in managing and preventing CDs (Table 4). 

**Table 4:** Prevalence of chronic disease according to sex. * Significant at p < 0.05 level.

Diseases	Male	Female	P-value	95% CI
	N (%)	N (%)		
Diabetes	14 (17.95)	56 (32)	0.002 *	2.55–4.67
Obesity	16 (20.51)	8 (4.57)	0.05	3.67-5.75
Hypertension	14 (17.95)	46 (26.29)	0.003*	4.78-6.54
Chronic kidney disease	4 (5.13)	3 (1.71)	0.04*	2.44-3.78
Cardiovascular disease	9 (10.39)	13 (7.43)	0.02*	3.12-4.67
Hyperlipidemia	12 (15.38)	22 (12.57)	0.003*	1.98-4.66
Asthma	3 (3.85)	7 (4)	0.03*	1.99-2.89
Breast cancer	0	4 (2.29)	0.03*	3.55-5.58
Thyroid cancer	0	1 (0.57)	0.04*	2.66-5.49
Cervical cancer	-	1 (0.57)	0.04*	1.45-2.99
Prostate enlargement	4 (5.13)	-	0.004*	2.66-2.95
Ovarian cancer	-	2 (1.14)	0.001*	2.89-3.58
Multiple sclerosis	0	2 (1.14)	0.004*	2.88-3.88
Hypothyroidism	2 (2.56)	10 (5.71)	0.002*	1.67-4.68
People with multimorbidity				
No chronic disease	42 (40.38)	191 (67.49)	0.002*	1.49-5.88
One chronic disease	35 (33.65)	59 (20.58)	0.03*	2.88-5.13
Two chronic diseases	11 (10.58)	17 (6.01)	0.02*	2.55-4.22
Three chronic diseases	08 (7.69)	7 (2.47)	0.04*	4.80-5.71
> Three chronic diseases	08 (7.69)	9 (3.18)	0.002*	5.87-7.82

## Discussion

This study provides an analysis of the prevalence of CD among adults in the Qassim region, highlighting significant differences based on age and sex. Understanding these patterns is crucial for developing effective public health interventions, particularly in light of the growing burden of non-communicable diseases in SA and the broader Middle East.

DM and hypertension

DM emerged as the most prevalent CD in our study, affecting n=70, 27.67% of participants, followed by hypertension at n=60, 23.72%. These findings are consistent with national and regional reports identifying DM and hypertension as leading health concerns in SA [19]. Notably, the higher prevalence of DM among women n=56, 32% compared to men n=14, 17.95% may be attributed to differences in healthcare-seeking behaviors, biological susceptibility, and lifestyle factors, as reported in similar studies from Riyadh and Jeddah [20, 21]. Interestingly, our results contrast with the study by Saquib N et al. [22], which found a higher prevalence of hypertension among men. This discrepancy may be due to variations in study populations, methodologies, and sample selection criteria.

Obesity and associated risk factors

Obesity is a major risk factor for CDs, contributing to an estimated 2.8 million deaths annually worldwide [23]. In our study, the prevalence of obesity was slightly higher in men n=16, 20.51% compared to women n=8, 4.57%, a trend that deviates from previous national reports where obesity was more common in women [24]. This unexpected trend may be influenced by cultural norms, physical activity disparities, and potential underreporting of obesity among women due to social stigma. Given that obesity is a well-established risk factor for diabetes and cardiovascular diseases, targeted lifestyle interventions are essential in addressing this growing public health issue [25].

Thyroid disorders and cancer

Hypothyroidism was significantly more prevalent among women n=10, 5.71%, a pattern well-documented in global research, where women are up to ten times more likely to develop thyroid disorders than men [26]. Additionally, the incidence of breast cancer among women in our study aligns with national data, reinforcing the need for increased awareness and early detection programs [27]. Increases in breast cancer incidence in SA and the Middle East have been linked to lifestyle factors such as delayed pregnancy, fewer children, reduced breastfeeding, physical inactivity, and high-calorie diets [28, 29, 30].

Multiple sclerosis

No available studies have reported multiple sclerosis as a chronic condition in the region. The prevalence of multiple sclerosis in our study was low, significantly lower than in other Gulf countries [31]. Only n=2, 0.79% of participants, all of whom were women, were affected. This relatively low prevalence of multiple sclerosis compared to other Gulf countries suggests potential underdiagnosis or differences in genetic predisposition [31].

Multimorbidity and aging

Multimorbidity, defined as the presence of two or more chronic conditions, was strongly associated with older age groups in our study. This finding aligns with previous studies from Canada and Riyadh, which have reported a significant increase in multimorbidity prevalence with age [32, 33]. As life expectancy continues to rise in SA, healthcare systems must adapt to address the growing burden of multimorbidity among older adults, emphasizing integrated care models and personalized management strategies.

Study limitations

Despite the strengths of our study, including its regional focus and detailed analysis, certain limitations should be acknowledged. The use of a convenience sampling method may limit the generalizability of our findings, and self-reported data could introduce recall bias. Future studies should incorporate probability-based sampling and incorporate objective health assessments such as clinical biomarkers to validate self-reported data and improve accuracy.

## Conclusions

We evaluated the prevalence of CDs among respondents from the Qassim region of SA. These results are consistent with prior research. However, larger surveys of this sort are necessary for effective policy formulation. Nonetheless, we recommend the implementation of comprehensive preventive programs that focus on health education and promote healthy lifestyles tailored to those most affected by CDs.
